# Supplementary material for: Identification of key regulators in prostate cancer from gene expression datasets of patients
Source: Sci Rep. 2019 Nov 11;9:16420. doi: 10.1038/s41598-019-52896-x (PMC6848149; doi:10.1038/s41598-019-52896-x)
Supplement: Supplementary file 1 — Supplementary Table 1. Top 103 hubs. [file 41598_2019_52896_MOESM1_ESM.docx]

**Identification of key regulators in prostate cancer from gene expression datasets of patients**

Irengbam Rocky Mangangcha^1,2,3,4^**^#^**, Md Zubbair Malik^4^**^#^**, Omer Kucuk^5^, Shakir Ali^1,2*^,

R. K. Brojen Singh^4*^

^1^School of Interdisciplinary Sciences and Technology, Jamia Hamdard,

New Delhi 110062, India

^2^Bioinformatics facility, BIF Jamia Hamdard & Department of Biochemistry, School of Chemical and Life Sciences &, New Delhi 110062, India

^3^Department of Zoology, Deshbandhu College, University of Delhi, New Delhi 110019, India

^4^School of Computational and Integrative Sciences, Jawaharlal Nehru University,

New Delhi 110067, India

^5^Emory Winship Cancer Institute, 1365 Clifton Road NE, Atlanta, GA 30322, USA

**^#^**Authors equal contribution

^*^Corresponding authors

To whom correspondence should be addressed.

E-mail: [brojen@jnu.ac.in](mailto:brojen@jnu.ac.in), [sali@jamiahamdard.ac.in](mailto:sali@jamiahamdard.ac.in)

**Supplementary Table 1**. Top 103 *hubs.*

| **Sl. No.** | ***Hub / Gene*** | **Degree**  **(*k*)** | **Sl. No.** | ***Hub / Gene*** | **Degree**  **(*k*)** | **Sl. No.** | ***Hub / Gene*** | **Degree**  **(*k*)** | **Sl. No.** | ***Hub / Gene*** | **Degree**  **(*k*)** |
| --- | --- | --- | --- | --- | --- | --- | --- | --- | --- | --- | --- |
|  | *CUL3* | 430 | **27.** | *ILF3* | 109 | **53.** | *CCT3* | 84 | **79.** | *CDKN2A* | 72 |
|  | *RNF2* | 304 | **28.** | *ILF2* | 109 | **54.** | *RPL23A* | 84 | **80.** | *RANBP2* | 72 |
|  | *TP53* | 290 | **29.** | *RPS2* | 108 | **55.** | *ADRB2* | 84 | **81.** | *KDM1A* | 72 |
|  | *CUL7* | 270 | **30.** | *AURKA* | 107 | **56.** | *GSK3B* | 83 | **82.** | *EEF2* | 72 |
|  | *CDK2* | 250 | **31.** | *RPL14* | 107 | **57.** | *RPL23* | 83 | **83.** | *RPL18* | 71 |
|  | *NPM1* | 231 | **32.** | *FBL* | 107 | **58.** | *RPS5* | 83 | **84.** | *CSNK2B* | 71 |
|  | *HNRNPU* | 202 | **33.** | *HSPA8* | 101 | **59.** | *RPL11* | 83 | **85.** | *RPS24* | 70 |
|  | *VCP* | 200 | **34.** | *RPS3* | 97 | **60.** | *RPL8* | 80 | **86.** | *MMS19* | 70 |
|  | *MYC* | 195 | **35.** | *RPS6* | 96 | **61.** | *RPSA* | 79 | **87.** | *ABCE1* | 70 |
|  | *HSP90AB1* | 173 | **36.** | *YWHAE* | 96 | **62.** | *USP7* | 79 | **88.** | *PLK1* | 69 |
|  | *HNRNPA1* | 162 | **37.** | *RPL5* | 92 | **63.** | *RPL15* | 79 | **89.** | *FBXO25* | 68 |
|  | *HDAC1* | 152 | **38.** | *C1QBP* | 92 | **64.** | *TRIM28* | 79 | **90.** | *NOP2* | 68 |
|  | *RPL35* | 143 | **39.** | *EZH2* | 91 | **65.** | *RPS7* | 78 | **91.** | *RPN1* | 67 |
|  | *VHL* | 140 | **40.** | *RAD23B* | 89 | **66.** | *RPL19* | 78 | **92.** | *FAF2* | 67 |
|  | *UBE2I* | 136 | **41.** | *NF2* | 88 | **67.** | *EIF3E* | 77 | **93.** | *RPL3* | 67 |
|  | *U2AF2* | 131 | **42.** | *PPP1CA* | 88 | **68.** | *PAXIP1* | 77 | **94.** | *CCT4* | 66 |
|  | *SUZ12* | 130 | **43.** | *RPLP0* | 88 | **69.** | *RACK1* | 76 | **95.** | *RPL22* | 66 |
|  | *YWHAQ* | 129 | **44.** | *PABPC1* | 87 | **70.** | *PPP1CC* | 75 | **96.** | *RPS18* | 66 |
|  | *TUBG1* | 121 | **45.** | *CLTC* | 87 | **71.** | *AHSA1* | 75 | **97.** | *TUBB* | 65 |
|  | *RPS8* | 120 | **46.** | *SNU13* | 87 | **72.** | *HDGF* | 75 | **98.** | *ECT2* | 65 |
|  | *NOP56* | 118 | **47.** | *RPS4X* | 87 | **73.** | *RPL10A* | 75 | **99.** | *HSPD1* | 65 |
|  | *CDK1* | 117 | **48.** | *HDAC2* | 87 | **74.** | *IGSF8* | 74 | **100.** | *RPS9* | 65 |
|  | *MRPL58* | 116 | **49.** | *AR* | 86 | **75.** | *RPL7A* | 74 | **101.** | *AURKB* | 65 |
|  | *SRP72* | 113 | **50.** | *EIF3A* | 85 | **76.** | *HIST1H3E* | 74 | **102.** | *RPL12* | 65 |
|  | *RPL6* | 113 | **51.** | *YWHAG* | 85 | **77.** | *RPS11* | 74 | **103.** | *RPL31* | 65 |
|  | *HSPA5* | 111 | **52.** | *NCL* | 85 | **78.** | *KPNB1* | 73 |  |  |  |
